# A Comparative Study of Mini-FLOTAC With Traditional Coprological Techniques in the Analysis of Cetacean Fecal Samples

**DOI:** 10.3389/fvets.2022.908486

**Published:** 2022-06-27

**Authors:** Federica Marcer, Rudi Cassini, Nancy Parisotto, Cinzia Tessarin, Erica Marchiori

**Affiliations:** Department of Animal Medicine, Production and Health, University of Padova, Legnaro, Italy

**Keywords:** cetacean, Mini-FLOTAC, copromicroscopy, sensitivity, helminths, Mediterranean Sea

## Abstract

Free-ranging cetaceans are considered sentinels for the marine ecosystem's health. New and non-invasive methods have been set up for the collection of fecal samples from free-ranging big whales at sea, permitting to gain an excellent epidemiological picture of parasitic infections in wild populations. To select the best protocol to be used for copromicroscopic examination in cetaceans stool samples, we evaluated the sensitivity of two commonly used techniques, i.e., a sedimentation-floatation method and the Mini-FLOTAC through validation by helminth isolation from the digestive tract. For this aim, gastrointestinal content and fecal samples were collected during necropsy from 44 cetaceans, including bottlenose dolphins (*Tursiops truncatus*), striped dolphins (*Stenella coeruleoalba*), sperm whales (*Physeter macrocephalus*), Risso's dolphins (*Grampus griseus*), Cuvier's beaked whales (*Ziphius cavirostris*), pilot whale (*Globicephala melas*), and fin whales (*Balaenoptera physalus*). Helminths were recovered through washing and filtering of the gastrointestinal contents and morphologically identified. Copromicroscopic examinations were performed on formalin-preserved fecal samples, using a sodium nitrate, sodium thiosulphate, and sucrose solution (s.g. = 1.450) for both methods. Helminths belonging to 9 taxa (i.e., the trematodes *Synthesium tursionis, Synthesium delamurei, Campula palliata, Braunina cordiformis, Pholeter gastrophilus*, the nematode *Anisakis* sp., cestodes of the family Tetrabothriidae and the acanthocephalan *Bolbosoma* sp.) were isolated. Eggs referable to the same taxa, with the exception of cestodes, were found in copromicroscopic analyses. Sensitivity of the Mini-FLOTAC method appeared higher or equal for all taxa, proving superior to the sedimentation-flotation method for the detection of all except *Anisakis* sp. The concordance of the two tests indeed revealed a moderate to perfect agreement (*kappa* values 0.42–1). Not excluding the limitations inherent to the techniques themselves, explanations for false-negative results at copromicroscopy could be linked to parasite-related factors, including prepatent infections, low parasitic burdens, or intermittent egg shedding. Notwithstanding these limitations, this study evidenced that the Mini-FLOTAC protocol approximates more accurately the composition of the gastrointestinal helminthic community of cetaceans from copromicroscopic examination, providing at the same time a quantitative estimation.

## Introduction

Free-ranging cetaceans, including large whales and dolphins, are considered sentinels for the marine ecosystem's health. Their long-life span, migratory habits, and role on the top of the trophic chain, as well as their potentiality to be carriers of anthroponotic pathogens, make them useful indicators of chemical pollution, ecological variations, and pressure of human activities on the marine environment (1–6). The increasing interest in the study of these species has brought the development of new methodologies for sampling live, free-ranging large whales, to obtain a reliable picture of the health of the wild population (4, 7). Nowadays, the greatest part of scientific data from large whales and cetaceans, in general, comes from the postmortem examination of stranded specimens, unavoidably falling into the bias caused by sampling mostly diseased animals. The same partiality applies to parasitological research, in that the collection of fecal samples from free-ranging, apparently healthy animals, would provide more valuable information about the health conditions of a population (8).

Gastrointestinal helminthofauna of odontocetes and mysticetes include nematodes, trematodes, cestodes, and acantocephalans. Diversity of the helminth community is a matter of interest for ecological studies on the host habitat distribution and local trophic web (9–12), and selected parasites species have been widely used as geographical tags in cetaceans (13, 14). Moreover, moderate to severe pathological patterns caused by parasitic infections are described in the hosts, especially when superinfected by selected parasites. The fluke *Pholeter gastrophilus* (Digenea: Heterophydae), has been associated with nodular fibrotic gastritis and alterations of gastric motility (15, 16). Flukes of the genus *Campula* (Digenea: Brachycladiidae) can cause chronic inflammation and hyperplasia of the pancreatic ducts in dolphins, affecting digestive and endocrine functions (17, 18). In several species of large and toothed whales, *Anisakis* spp. (Nematoda: Anisakidae) is responsible for ulcers and gastritis as recently reported by Pons-Bordas et al. (2020) (19) and Hrabar et al. 2021 (20) in the Atlantic and the Pacific Ocean, and finally, acantocephalans of the genus *Bolbosoma* (Acanthocephala: Polymorphidae) are associated to chronic, ulcerative to perforated enteritis (21, 22).

Copromicroscopic examination of cetacean feces, following fecal samples collection *in vivo*, are reported in the literature, using different quali-quantitative techniques, and several different taxa, including helminths and protozoa, have been successfully detected by these methods (4, 6, 7, 23, 24). Nevertheless, their reliability has never been verified through comparison with a reference test. To fill this gap, in this study the performances of two copromicroscopic methods, i.e., a classical sedimentation-flotation method and the Mini-FLOTAC, are assessed by comparison of their results with the isolation of helminths from the gastrointestinal tract.

## Materials and Methods

In the period between 2009 and 2020, the gastrointestinal tract of 44 cetaceans, including toothed (*n* = 39) and baleen whales (*n* = 5), stranded dead along the Italian coastlines (Tyrrhenian, Ligurian, and the Adriatic Sea), were analyzed to detect helminth parasites in the digestive system (Table 1). After opening gastric chambers, the content was collected and the mucosa was analyzed for parasites and nodular lesions. The entire intestine, or selected portions in the case of large whales (small and gross intestine, at least 10 m each), was opened for parasitological examination. Gastric and intestinal contents were separately filtered with 1.0 and 0.5 mm mesh sieves for the isolation of helminths. When possible, the pancreas and liver of the same animals were collected as well and analyzed for the presence of trematode parasites through washes of organs slices (1 cm thick) in tap water. All parasites recovered were washed in physiological saline, counted and fixed in 70% ethanol, and subsequently identified at the stereomicroscope or optic microscope by their morphometric features, following specific literature (25, 26). Fecal samples were collected from the rectum of all animals and a copromicroscopic exam was performed on the stool. Part of the rectal content (about 10 grams) was also preserved in 5% formalin. Subsequently, one aliquot of each fecal sample was centrifuged under the fume hood for the elimination of storage liquid and processed for qualitative examination by i) a sedimentation-flotation (SF) and ii) Mini-FLOTAC technique in combination with Fill-FLOTAC (MF). Briefly, for the first method, 2–3 grams were homogenized in a high gravity solution (sodium nitrate, sodium thiosulphate, and sucrose, specific gravity = 1.450) and centrifuged at 2,000 rpm for 5 min in a 5 ml tube. A coverslip was put on the top of the tube for recovering parasitic elements through observation at the optic microscope at 10–20X magnification. The remaining part of the aliquot (2 grams), was processed through a Mini-FLOTAC apparatus combined with Fill-FLOTAC, using the same high s.g. solution as in the first method to obtain a 1:10 dilution (27). Helminths' eggs were identified following literature to the lowest possible taxonomic level as morphological features allowed, i.e., to species level for *Pholeter gastrophilus* (Trematoda: Heterophyidae) and *Braunina cordiformis* (Trematoda: Brauninidae), at the genus level for *Ogmogaster* (Trematoda: Notocotylidae), *Anisakis* (Nematoda: Anisakidae) and *Bolbosoma* (Acantocephala: Polymorphidae), while eggs of flukes of the family Brachycladiidae were not distinguished to lower levels (25, 28).

**Table 1:** Sampled animals listed by species, conservation condition of the carcasses, and number of sampled organs.

**Species**	**N**	**CC**	**GI**	**LP**
*Tursiops truncatus*	19	1–3/4	19	15
*Stenella coeruleoalba*	7	2	7	4
*Physeter macrocephalus*	7	1–3	7*	6
*Grampus griseus*	4	1–2	4	1
*Ziphius cavirostris*	1	3/4	1	0
*Globicephala melas*	1	Nd	1	1
*Balaenoptera physalus*	5	1–3	5*	4

*N, total number; CC, conservation condition code (sensu Geraci and Lounsbury, 2005); GI, gastrointestinal tract, considering also fecal sample; LP, liver, and pancreas*.

*Portions of the intestinal tract, 20 meters at least from the small and gross intestine.

For the comparison of the two diagnostic methods, results from the MF technique were reduced to qualitative statements, i.e., positive or negative. Concordance of the two copromicroscopic tests was calculated as the number of samples with the same result out of the total number of samples examined (% concordance). This was further evaluated using kappa-type statistics (29), which express the proportion of agreement determined beyond chance with a value (parameter *k*) of 0 (no agreement) to 1 (perfect agreement). Results of the two copromicroscopic exams were then compared with the isolation of helminths from the gastrointestinal system, to define which test had the highest sensitivity for the detection of each taxon. The specificity of the two tests was assumed to be 100%. Analysis of the sensitivity of the two tests was performed using the online epidemiological calculator software EpiTools (http://epitools.ausvet.com.au).

## Results

**Figure 1:**
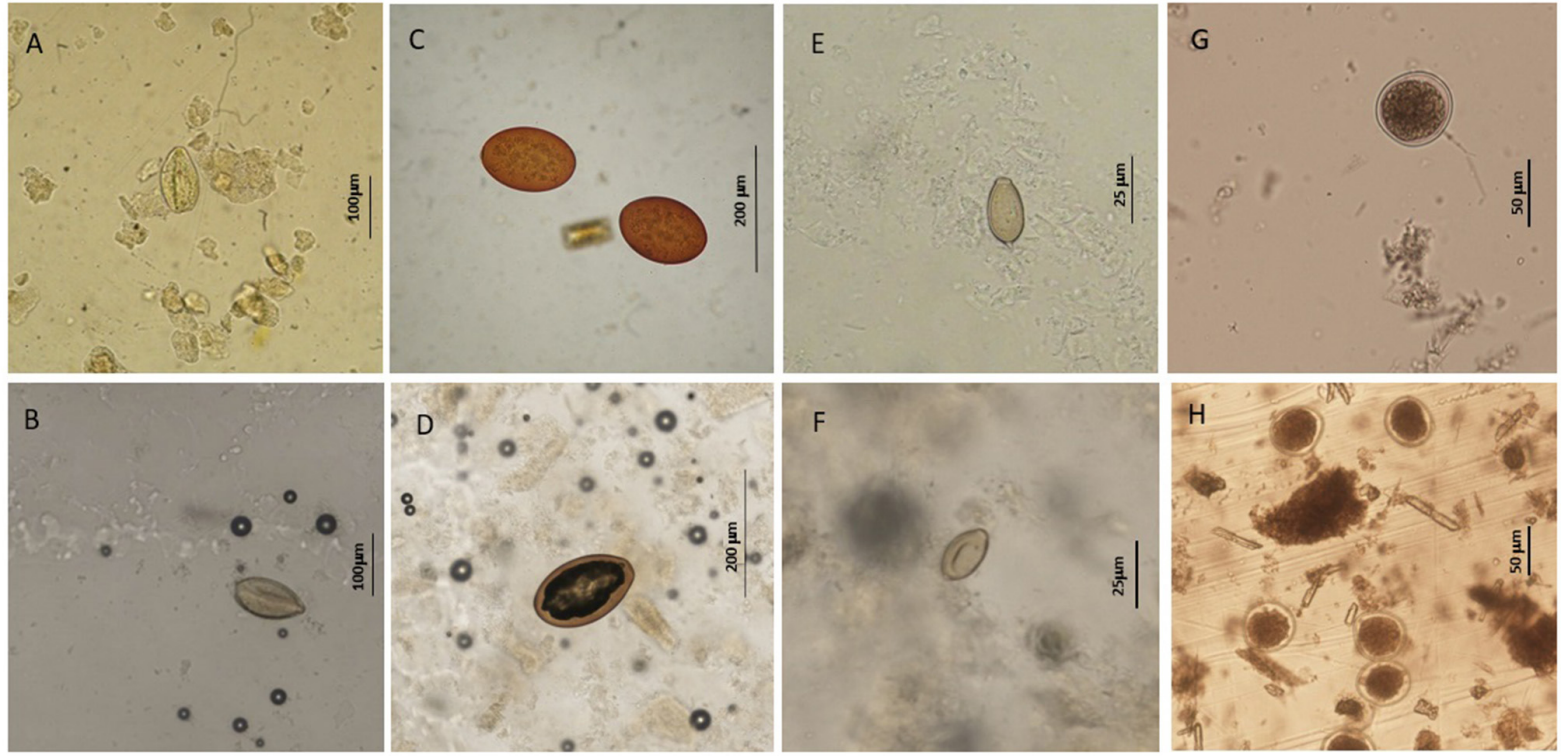
Helminth parasites' eggs detected in this study by sedimentation and flotation method **(A,C,E,G)** and Mini-FLOTAC technique **(B,D,F,H)** using fresh and preserved samples respectively. **(A,B)** eggs of *Synthesium tursionis* and **(C,D)** eggs of *Braunina cordiformis* from *Tursiops truncatus*; **(E,F)** eggs of *Pholeter gastrophilus* from *Stenella coeruleoalba*; **(G,H)** eggs of *Anisakis* sp. from *Stenella coeruleoalba*.

**Figure 2:**
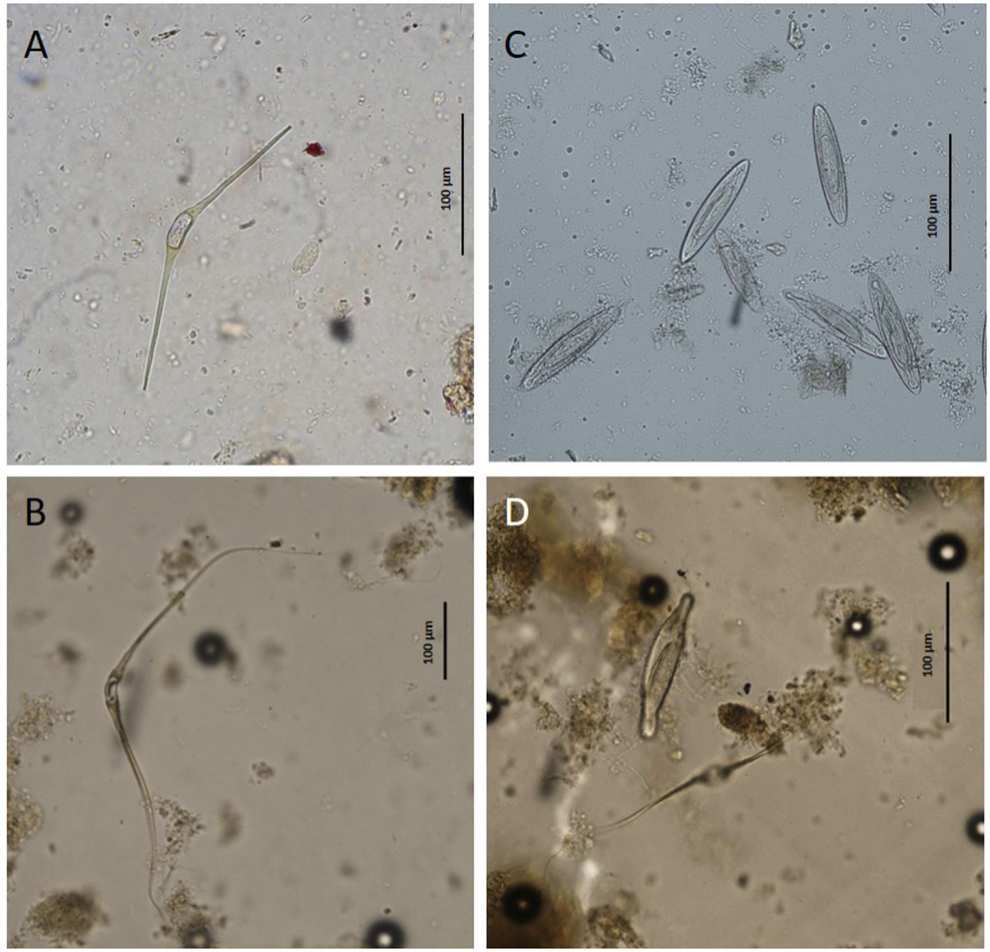
Eggs of *Ogmogaster antarcticus* **(A,B)** and *Bolbosoma* sp. **(C,D)** from *Balaenoptera physalus* obtained by sedimentation and flotation method **(A,C)** and Mini-FLOTAC technique **(B,D)**.

Specimens of trematodes belonging to the families Brachycladiidae (*Synthesium tursionis, Synthesium delamurei, Campula palliata*), Brauninidae (*Braunina cordiformis*) and Heterophyidae (*Pholeter gastrophilus*), nematodes of the Anisakidae (*Anisakis* sp.), cestodes (Tetrabothriidae and plerocercoid larvae of Tetraphyllidea) and acanthocephalans of the genus *Bolbosoma* were recovered from the digestive tract of toothed whales, while trematodes belonging to the family Notocotylidae (*Ogmogaster antarcticus*), cestodes (Tetrabothriidae, *Tetrabothrius ruudi*) and the acanthocephalans *Bolbosoma balenae* and *Bolbosoma* sp. were found in fin whales. No eggs attributable to additional taxa neither eggs nor proglottids of cestodes were recovered during copromicroscopic observations. Figures 1, 2 show the morphological features of the eggs evidenced by the two copromicroscopic methods and well-maintained morphology after storage in formalin.

Considering positivity at helminth isolation and/or one of the two copromicroscopic tests, an overall percentage of 86.4 % of the animals (38/44) proved positive for at least one parasitic taxon and 52.3% (23/44) showed co-infection by more than one parasitic species. Flukes of the family Brachycladiidae had the highest prevalence among odontocetes (41%, 16/39), while in mysticetes *Ogmogaster* sp. had a prevalence of 100% (5/5).

*K*-values for analysis of the concordance of the two copromicroscopic tests ranged from 0.42 (moderate agreement) for the detection of eggs of *B. cordiformis* to 1 (perfect agreement) for eggs of *Anisakis* sp (Table 2). Evaluation of the overall number of taxa detected with the two techniques showed that in 32/44 cases the same parasitic taxa were found by both tests, while MF found a wider range of parasitic taxa in 10/44 samples. Conversely, the SF method detected a higher number of parasitic taxa in 2/44 cases with respect to MF.

**Table 2:** Concordance of the SF and MF methods for the detection of the helminthic taxa.

**Phylum/Class**	**Family**	**Taxon**	**Concordance (%)**	***Parameter k***
Trematoda	Brachycladiidae	*Synthesium/Campula* spp.	88.6	0.69
	Heterophyidae	*P. gastrophilus*	97.7	0.84
	Brauninidae	*B. cordiformis*	86.3	0.42
	Notocotylidae	*Ogmogaster* sp.	93.2	0.54
Nematoda	Anisakidae	*Anisakis* spp.	100	1
Acantocephala	Polymorphidae	*Bolbosoma* spp.	97.7	0.66

The analysis of the sensitivity of the two copromicroscopic tests showed higher sensitivity of MF for all taxa, except *Anisakis* sp. for which both had the same performance (sensitivity 56%). Sensitivity of 0% was registered using both methods for the research of cestode eggs.

Remarkably, 12 animals were positive during the copromicroscopic examination while their postmortem examination was negative for the corresponding taxa. In particular, MF was positive for 5 different taxa in 8 animals without correspondence with the isolation from the gastrointestinal tract and the same occurred with the SF technique in 4 animals (Table 3).

**Table 3:** Comparison of the results of SF and MF methods with isolation of corresponding helminthic taxa from the stomach/intestine and calculation of their sensitivity.

	**Helminth isolation from GI**		**SF (pos/tot)**	**MF (pos/tot)**	**Se SF (%) (CI 95%)**	**Se MF (%) (CI 95%)**
Brachycladiidae	pos	13	6/13	10/13	46 (19–74)	76 (46–95)
	neg	31	2/31	3/31		
*P. gastrophilus*	pos	13	3/13	4/13	23 (5–54)	31 (10–61)
	neg	31	0/31	0/31		
*B. cordiformis*	pos	9	4/9	5/9	44 (14–78)	56 (21–86)
	neg	35	1/35	2/35		
*Ogmogaster* sp.	pos	2	1/2	2/2	50 (1–98)	100 (16–100)
	neg	42	1/42	3/42		
Cestodes	pos	8	0/8	0/8	0 (0–37)	0 (0–37)
	neg	36	0/36	0/36		
*Anisakis* spp.	pos	15	9/15	9/15	56 (30–80)	56 (30–80)
	neg	29	0/29	0/29		
*Bolbosoma* spp.	pos	3	1/3	2/3	33 (1–90)	67 (0.9–99)
	neg	41	0/41	0/41		

*Se, sensitivity; SF, Sedimentation-Flotation method; MF, Mini-FLOTAC method*.

## Discussion

Different qualitative copromicroscopic methods have been used in literature for the examination of cetacean fecal samples, with the occasional use of quantitative techniques for the estimation of parasitic burden in free-ranging large whales (23). Nevertheless, a comparison of the performances of copromicroscopic techniques applied to cetacean stool samples has never been conducted, though the ability of Mini-FLOTAC to detect protozoan cysts has already been reported in stool samples of live, free-ranging large whales (6). In this study, the direct isolation of helminths from the digestive system after the necroscopic examination was used as a standard reference for the comparison of the results of two tests to be used *in vivo* in future sampling. Adult specimen isolation is considered the most sensitive and reliable method for the description of the helminthic community, but the collection of such data can only be achieved after necropsies of stranded animals, which prove thus essential in this preliminary step to set up methods to be applied *in vivo*. Nevertheless, specimens that were positive for helminthic taxa at copromicroscopic exams while negative at post-mortem examination highlighted the absence of a true gold standard diagnostic test, probably due to limitations associated with all techniques. Bad preservation conditions of the animals could hamper the detection of tiny parasites during the necropsy, and the process of washing and filtering gastrointestinal content may also have altered the integrity of parasites. On the other side, copromicroscopic examination can be falsely negative in case of prepatent infections, single-sex helminth infections for species with separate gender, intermittent egg shedding, or parasites producing low numbers of eggs (lower than 5 eggs per gram for MF) or for low parasitic burdens. In the case of *P. gastrophilus*, the host's immune response reacts to the adult parasites by forming fibrotic nodules into the gastric wall in an attempt to isolate them. These nodules are generally connected with the gastric lumen by narrow conducts through which the eggs are shed. The interaction between the host's immune response and parasites affects the architecture of the nodules, as well as their connection with the gastric lumen for eggs emission (30, 31). In some cases, only a large amount of eggs, grouped into large masses, were detected within the gastric fibrotic nodules, in absence of adult worms, as already observed (16). These eventualities may affect eggs shedding in the feces, making copromicroscopy a non-suitable method for the diagnosis of the infection. As for cestodes, no eggs nor mature proglottids were detected in the fecal samples of animals harboring parasites in the intestine. The distribution of gravid proglottids in the intestine and in the feces is irregular for parasites of the order Cyclophillidea (32), making it challenging to detect the elements in a single stool sample. Moreover, the type of high s.g. solution can affect the sensitivity of the copromicroscopic method for cestode eggs suggested for terrestrial mammals (33, 34). The concentration of the feces starting from the larger amount of fecal material, and the use of different types of floatation solutions should be evaluated for future studies on copromicroscopic research of cestode eggs in cetacean feces.

Due to the probability of false-negative results at all tests, the highest accuracy in the estimation of the composition of gastrointestinal helminthic fauna in these species can only be achieved by performing both kinds of research, i.e., of adult helminths in the digestive system and of the resistant elements in the feces.

Based on the results of this first survey, MF proved better than the SF method to approximate the helminthic fauna composition from fecal examination, having higher or equal sensitivity for all taxa. The low prevalence of some parasitic taxa and the number of sampled animals may represent a limit in the interpretation of sensitivity results. Unavoidable constraints in obtaining samples from these free-ranging animals come from the impossibility of planning a sampling campaign for such species. Sharing this protocol among different geographical areas will permit not only to increase the number of samples but also investigate the sensitivity of the test toward other helminthic taxa.

A comparison of the two copromicroscopic tests showed a generally moderate to good concordance. The highest agreement between the two was calculated for the detection of *Anisakis* sp. eggs, this being the taxon for which the highest sensitivity of SF was found. The large amount of eggs generally produced by nematodes of the superfamily Ascaridoidea (35) may enhance the probability of detecting the eggs at copromicroscopy. Actually, both MF and SF methods provided false-negative results when exclusively larvae or immature specimens of *Anisakis* sp. were detected in the stomach of the animals, allowing us to infer a sensitivity of 100% for both test for *Anisakis* sp. in the case of patent infections.

The FLOTAC and mini-FLOTAC techniques have been validated for use in humans, as well as in domestic and wild terrestrial animals, demonstrating higher diagnostic sensitivity and accuracy when compared to other traditional techniques for the detection of intestinal parasitic infections (36–43). Preservation of samples in 5% formalin seems not to affect the sensitivity of MF (33, 42, 44) and offers at the same time the opportunity to process the samples after weeks from the collection (27). In this study, the examination of stored fecal samples was carried out after a much longer time (several months or years) virtually limiting the sensitivity of MF, which was anyway higher for MF than for the SF method. The morphology of the eggs appeared maintained in comparison with freshly collected samples. Nevertheless, a more detailed vision of eggs was sometimes observed through the SF technique, being less affected by the quality of fecal samples in terms of debris content (Figures 1B,F,H). A higher dilution rate of the sample could solve this issue as suggested by Cringoli et al. (27), decreasing the quantity of fecal debris. Finally, different types of high gravity solutions should also be tested to unveil differences in the sensitivity of the two tests, considering that selected taxa preferably float with specific solutions (27).

The results obtained with this study highlight the greater sensitivity of MF compared to the SF method for the detection of eggs of helminth parasites residing in the digestive system of cetaceans. Reliability of this comparison is permitted by the execution of necropsies and detection of helminth parasites in the digestive tract of the same animals. A wider sampling and the use of different floatation solutions, comparing different storage methods, could be useful to optimize the examination protocol. The correlation between fecal egg burden and intensity of infection deserves as well a wider sampling effort to draw significant conclusions since the number of positive animals for the taxa here considered is insufficient for a proper statistical study.

In conclusion, the MF method can be successfully used to study the parasitofauna of free-living marine mammals obtaining at the same time the estimation of parasitic burden. This may also prove useful for managing parasitic infections in captivity (45) with the proper use of antihelminthic treatments in selected cases.

## Data Availability Statement

The original contributions presented in the study are included in the article/Supplementary Material, further inquiries can be directed to the corresponding author.

## Author Contributions

FM and EM performed the experimental conception and design and collected the samples. FM performed isolation and identification of helminth parasites. EM performed the statistical analysis and wrote the manuscript. RC reviewed the statistical analysis design. NP and CT performed copromicroscopic examinations. All Authors participated in the revision of the manuscript. All authors contributed to the article and approved the submitted version.

## Conflict of Interest

The authors declare that the research was conducted in the absence of any commercial or financial relationships that could be construed as a potential conflict of interest.

## Publisher's Note

All claims expressed in this article are solely those of the authors and do not necessarily represent those of their affiliated organizations, or those of the publisher, the editors and the reviewers. Any product that may be evaluated in this article, or claim that may be made by its manufacturer, is not guaranteed or endorsed by the publisher.
