# Supplementary material for: A Comparative Study of Mini-FLOTAC With Traditional Coprological Techniques in the Analysis of Cetacean Fecal Samples
Source: Front Vet Sci. 2022 Jun 27;9:908486. doi: 10.3389/fvets.2022.908486 (PMC9271992; doi:10.3389/fvets.2022.908486)
Supplement: Supplementary file 1 [file Table_1.DOCX]

**Supplementary data**

Table 1. List of anamnestic data of animals included in the study, helminth taxa detected by the two copromicroscopic techniques and by isolation from the digestive system.

|  | **Species** | **Stranding** | **Code** | **SF** | **MF** | **GI** | **LP** |
| --- | --- | --- | --- | --- | --- | --- | --- |
| 1 | *Tursiops truncatus* | Jul- 09 | ND | neg | neg | neg | neg |
| 2 | *Tursiops truncatus* | Jul- 09 | 3/4 | neg | Bra, Bc | Bra | neg |
| 3 | *Tursiops truncatus* | Jul- 09 | 2 | Bra | Bra | Bra | neg |
| 4 | *Stenella coeruleoalba* | Sept- 09 | 2 | neg | Bra, Pg | Pg | Bra |
| 5 | *Physeter macrocephalus* | Dec-09 | 1 | A | A | A* | neg |
| 6 | *Physeter macrocephalus* | Dec-09 | 1 | A | A | A* | neg |
| 7 | *Balaenoptera physalus* | Jan-11 | 2 | neg | O | O* | neg |
| 8 | *Tursiops truncatus* | May-11 | 2 | neg | neg | Bra, Pg, Bc, A | neg |
| 9 | *Tursiops truncatus* | Jul-11 | 2 | A | Bra, A | Bra, A | neg |
| 10 | *Balaenoptera physalus* | Oct-11 | 2 | neg | O | neg* | neg |
| 11 | *Balaenoptera physalus* | Oct-11 | 1 | neg | O, Bo | Cest, Bo* | neg |
| 12 | *Stenella coeruleoalba* | Jan-12 | 2 | neg | Bra | Pg, Cest, A | nd |
| 13 | *Grampus griseus* | Jan-12 | 2 | Bra | Bra | Bra | neg |
| 14 | *Stenella coeruleoalba* | Jan-12 | 2 | neg | neg | Cest, A | neg |
| 15 | *Stenella coeruleoalba* | Jan-12 | nd | A | A | Cest, A, Bo | neg |
| 16 | *Ziphius cavirostris* | Jan-12 | 4 | neg | neg | neg | nd |
| 17 | *Stenella coeruleoalba* | Mar-12 | nd | neg | neg | A | nd |
| 18 | *Grampus griseus* | Apr-12 | 1 | neg | neg | Cest | nd |
| 19 | *Grampus griseus* | Apr-12 | 2 | neg | neg | neg | nd |
| 20 | *Grampus griseus* | Jun-12 | 1 | neg | neg | neg | nd |
| 21 | *Globicephala melas* | May-13 | nd | neg | neg | neg | neg |
| 22 | *Tursiops truncatus* | Oct-13 | 3/4 | neg | neg | Bra, Pg | nd |
| 23 | *Tursiops truncatus* | May-14 | 2 | Bra | Bra, Bc | Bra, Bc | neg |
| 24 | *Stenella coeruleoalba* | Jun-14 | nd | neg | neg | Bc, A | neg |
| 25 | *Tursiops truncatus* | Jun-14 | ND | Bra, Bc | Bra, Bc | Bra, Bc | neg |
| 26 | *Physeter macrocephalus* | Sept-14 | 2 | A | A | A* | neg |
| 27 | *Physeter macrocephalus* | Sept-14 | 2 | A | A | A* | neg |
| 28 | *Physeter macrocephalus* | Sept-14 | 2 | A | A | A* | neg |
| 29 | *Tursiops truncatus* | Oct-14 | 1 | Bra | Bra | Bra, Pg | neg |
| 30 | *Balaenoptera physalus* | Jan-15 | 3 | O | O | O* | neg |
| 31 | *Tursiops truncatus* | Feb-15 | 2 | neg | neg | neg | neg |
| 32 | *Tursiops truncatus* | May-15 | 2 | Bra | Bra | Pg, Cest | nd |
| 33 | *Stenella coeruleoalba* | Apr-2016 | 2 | Bra | Bra | Pg, Cest | neg |
| 34 | *Physeter macrocephalus* | May-2016 | nd | A | A | A, Cest* | neg |
| 35 | *Physeter macrocephalus* | Oct-2016 | 3 | A | A | A* | nd |
| 36 | *Tursiops truncatus* | Jun-17 | 3 | Pg | Bra, Pg, Bc | Bra, Pg, Bc | neg |
| 37 | *Tursiops truncatus* | Jul-17 | 3 | neg | neg | Pg | neg |
| 38 | *Tursiops truncatus* | Jul-17 | nd | Bra | Bra, Bc | Bra** | neg |
| 39 | *Tursiops truncatus* | Aug-17 | 3 | neg | neg | Pg, Bc | neg |
| 40 | *Tursiops truncatus* | Sept-17 | nd | Bc | neg | Pg | neg |
| 41 | *Tursiops truncatus* | Jan-19 | 2 | Bc | Bc | Bra, Bc, A | neg |
| 42 | *Balaenoptera physalus* | Nov-20 | 3 | O, Bo | O, Bo | Bo* | nd |
| 43 | *Tursiops truncatus* | Jun-20 | nd | Pg, Bc | Pg | Pg, Bc | nd |
| 44 | *Tursiops truncatus* | Jul-20 | nd | Pg, Bc | Pg, Bc | Pg, Bc | nd |

Bc, *Braunina cordiformis*; Pg, *Pholeter gastrophilus*; A, *Anisakis* sp; Bra, Brachycladiidae; Bo, *Bolbosoma* sp.; O, *Ogmogaster* sp. SF, sedimentation-flotation technique, MF, MiniFLOTAC technique. Code, conservation condition code of the carcass (*sensu* Geraci & Lounsbury, 2005); GI, gastrointestinal tract; LP, liver and pancreas; nd, not determined. *selected portions of small and gross intestine were analysed; **Stomach not analysed.
